# The alteration and potential relationship of vaginal microbiota and chemokines for unexplained recurrent spontaneous abortion

**DOI:** 10.1097/MD.0000000000023558

**Published:** 2020-12-18

**Authors:** Tao Fan, Xing-Ming Zhong, Xiang-Cai Wei, Zhu-Lin Miao, Si-Ying Luo, Heng Cheng, Qing Xiao

**Affiliations:** a Department of Obstetrics, The Eighth Affiliated Hospital, Sun Yat-sen University, Shenzhen, 518003; b Department of Obstetrics, Guangdong Provincial Family Planning Institute, Guangzhou, 510080; c Department of Obstetrics, Guangdong Provincial Maternal and Child Health Hospital, Guangzhou, 511400, China.

**Keywords:** 16S rRNA, URSA, vaginal flora, vaginal secretion

## Abstract

The diagnosis and treatment of unexplained recurrent spontaneous abortion (URSA) is an important and hot topic in the field of obstetrics and gynecology. During our clinical investigation (observation), we have found that URSA patients usually experience recurrent vaginitis or vaginal dysbacteriosis during periods of non-pregnancy, pregnancy, and post-abortion. However, there is no research on vaginal dysbacteriosis's influence on URSA. Using women with normal induced abortion as a control group, and using 16S rRNA sequencing, which helps to screen differentially expressed flora, this study discusses the relevance between differential bacteria at the genus level and the incidence of URSA. Another aim of this study is to determine whether certain pathogenic genera can cause an imbalance in immune tolerance of the maternal and fetal interface through regulatory chemokines, which leads to recurrent spontaneous abortion. This article has explored URSA pathogenesis from the perspective of differentially expressed vaginal flora, which has great theoretical significance for the early diagnosis and treatment of URSA.

## 1 Introduction

**Figure 1:**
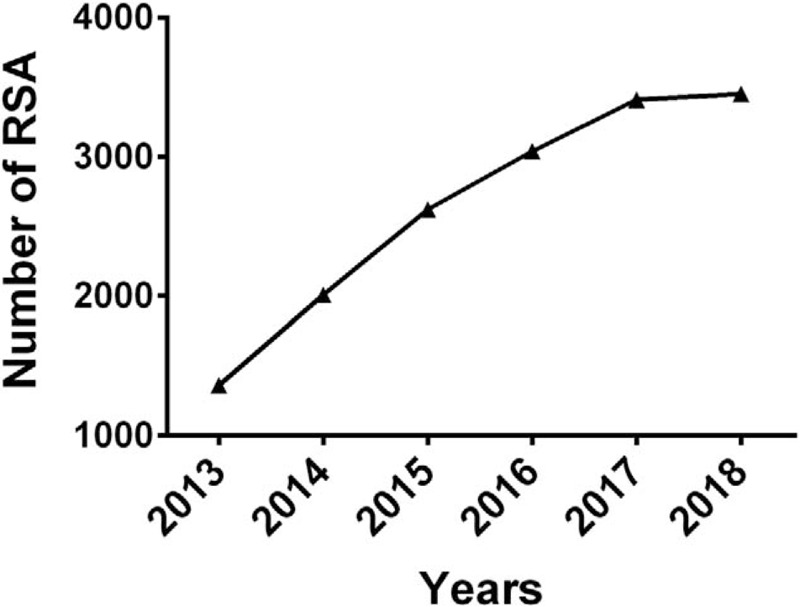
RSA patients from Guangzhou Women and Children's Medical Center in recent 5 years.

Recurrent spontaneous abortion (RSA) is a severe disease with a potential negative influence on women's health. Epidemiological research has shown that, after 2 clinical abortions, the pregnancy abortion risk is then approximately 24%; this number increases to 30% after 3 abortions and to 40% after 4 abortions.^[1,2]^ We have counted the number of patients at the Women and Children's Hospital who experience RSA every year and found that, within the past 5 years, the outpatient rate of RSA has continued to rise, with 41.83% of new patients on average every year (Fig. 1). In half of these patients, RSA causes are unexplained, making diagnosis and treatment difficult. Many couples of childbearing age cannot have their own children due to repeated abortions, which has serious consequences on both their families and society. In view of this, we conducted initial research on unexplained recurrent spontaneous abortion (URSA) causes to determine its pathogenesis and provide a reference for clinical application.

RSA depends on many factors^[1,4,5]^ including chromosomes,^[6,7]^ heredity,^[3,8]^ dissection,^[9]^ the endocrine system,^[10]^ placental abnormality,^[11]^ infection,^[12]^ immunity,^[2,13]^ thrombosis,^[2,8,14]^ and the environment,^[15]^ among others. Among all the cases, 50% still cannot be explained.^[3,16]^ Based on research statistics, and observations from long-term clinical studies, researchers have found that URSA patients usually experience recurrent vaginitis or vaginal dysbacteriosis during the periods of non-pregnancy, pregnancy, and post-abortion. According to the statistics collected from 4483 URSA patients, 136 were diagnosed with vaginitis, which accounted for 3.03% of all URSA patients. Therefore, it is necessary to determine whether vaginal dysbacteriosis contributes to URSA.

Bacterial vaginosis (BV) is a disorder of the vaginal micro-ecosystem, which seems related to the increased the risk of abortion,^[17]^ premature delivery,^[18]^ and puerperal endometritis.^[19]^ BV accounts for 50% of all types of vaginitis.^[20]^ According to previously published research, using Papanicolaou staining and Gram staining to observe BV-related bacteria involves cultivating Gardnerella to diagnose BV in women of childbearing age who have a history of spontaneous abortion (N = 30) and RSA (N = 31). BV is frequently found (*P* < .05) in women who have a spontaneous abortion in the middle and late periods of pregnancy, but no significant correlation has been found between BV and RSA (*P* > .05). Among all 61 women of childbearing age, 47 (77%) experienced abortion during early pregnancy (≤14 weeks) and 14 (23%) experienced abortion during middle pregnancy (14–28 weeks); therefore, BV and middle pregnancy abortion are significantly correlated (*P* < .05).^[21]^ However, according to Ralph, who used Gram staining of a vaginal smear to detect BV and evaluated the influence of BV on the pregnancy and early pregnancy abortion rates, there was no difference in the pregnancy rate between BV patients and women with normal vaginal flora. In contrast, BV was related to an increased risk of early pregnancy abortion.^[22]^ According to a meta-analysis, some studies have evaluated BV as a risk factor that influences abortion. Among all 20,232 patients, it was found that BV could significantly increase the risk of spontaneous abortion (odds ratio, 9.91; 95% CI, 1.99–49.34) and maternal infection (odds ratio, 2.53; 95% CI, 1.26–5.08).^[23]^ By using denaturing gradient gel electrophoresis, combined with sequencing, to perform a diversity analysis of the vaginal flora of both BV patients and healthy women, it was found that the main bacterial categories include Firmicutes, Bacteroidetes, Actinobacteria, and *Fusobacteria*. Three categories (*Bacteroidetes*, Actinobacteria, and *Fusobacteria*) and 8 genera (*Gardnerella*, *Atopobium*, *Megasphaera*, *Eggerthella*, *Aerococcus*, *Leptotrichia/Sneathia*, *Prevotella*, and *Papillibacter*) are closely related to BV and, thus, can be used as potential clinical diagnostic targets.^[24]^ The studies above have demonstrated that vaginal dysbacteriosis is significantly correlated with spontaneous abortion.

Lower genital tract infection is one cause of abortion. Most intra-amniotic infections appear to be caused by microorganisms entering the amniotic fluid from the lower genital tract (vagina or cervix). The changing vaginal micro-ecosystem is related to intrauterine ascending infections.^[25,26]^ By producing methionine proteins that activate complement with amino terminal N-formylation and chemokines, the bacteria can increase neutrophils’ ability to reach the infection sites.^[27]^ These inflammatory factors can activate immune cells of the endometrium, which leads to invasion by the trophoblast cells due to the maternal immune response; this can further cause some recurrent early–middle pregnancy abortions. The balance between the immune response and tolerance is the basis of fetal survival. The immune tolerance of the maternal–fetal interface will be broken by the partial dysfunction of immune cells and abnormal cytokine secretion, which leads to spontaneous abortion.^[28,29]^ Between 10% and 15% of abortions are caused by embryo–fetus infection. However, this may be a low estimate since the causes of sub-clinical abortion have yet to be found. Diagnosis of septic abortion is only based on histological examination of the fetus and placental tissue or through retrospective analysis of an isolated culture or genome detection of suspicious infectious agents.^[30]^ Actually, acute infection may occur as a result of all microorganisms, which may occasionally cause abortion, but only a very limited number of them can induce the chronic maternal diseases that may cause RSA.^[30]^ In recently, Ding et al provide experimental evidence that supports vaginal flora dysbiosis in women with RM.^[31]^

Therefore, it is necessary to determine which infections of microorganism induce RSA. Many abortions caused by infections lack effective methods for early diagnosis, and the mechanism behind RSAs caused by vaginal dysbacteriosis has yet to be reported. Based on the research above, we propose that pathogenic flora, which is translocated into the uterus, causes infection through the vagina, inducing the local immune response by activating chemokines. This causes local immune microcirculation disturbance, which leads to RSAs. Here, we screened the pathogenic flora by analyzing the URSA vaginal flora's expression flora and studying the regulatory mechanism to provide a new method for detecting URSA pathogenesis. The abnormal expression of vaginal flora may become a new target, which can provide important theoretical guidance for further advancement of URSA diagnosis and treatment.

## 2 Materials and methods

### 2.1 Subjects and sample collection

This study included 31 patients with URSA who were admitted to our hospital from January 2010 to December 2016 and 27 patients with normal induced abortion who served as controls. Inclusion criteria of the RSA group are:

(1) no abnormalities detected in hepatitis B surface antigen (HbsAg), hepatitis C virus (HCV), human immunodeficiency virus (HIV), human papilloma virus (HPV), or human cytomegalovirus (HCMV);
(2) no medical complications, normal blood group antibody titer, anti-sperm antibody-negative, anti-cardiolipin antibody-negative, and anti-endometrial antibody-negative;
(3) normal chromosome number and structure in both parents, and normal chromosomes in abortion villus samples;
(4) Two or more spontaneous abortions, current pregnancy, or menopause period <3 months, in those receiving treatment for miscarriage prevention;
(5) voluntary participation in the “Guangzhou Women and Children's Medical Center vaginal flora sample library and patient clinical database” of patients who provided signed informed consent.

Inclusion criteria for the normal induced abortion group are:

(1) no abnormalities detected in hepatitis B surface antigen (HbsAg), HCV, HIV, HPV, or human cytomegalovirus (HCMV);
(2) no medical complications, normal blood group antibody titer, anti-sperm antibody-negative, anti-cardiolipin antibody-negative, and anti-endometrial antibody-negative;
(3) no history of spontaneous abortion, current pregnancy, or menopause period less than 3 months, without treatment for miscarriage prevention;
(4) voluntary termination of pregnancy;
(5) voluntary participation in the “Guangzhou Women and Children's Medical Center vaginal flora sample library and patient clinical database” of patients who provided signed informed consent.

The ethics committee of the eighth hospital affiliated of Sun Yat-Sen University and Nanhai Hospital of Southern Medical University (NO. LLPJ201801) reviewed this study.

The vagina was opened with a vaginal speculum, and secretions from the posterior vaginal vault were obtained with a sterile cotton swab. After the material was collected, the sterile cotton swab was immediately placed in a protective solution, and the sample was stored in a sealed state at 4 to 8 °C. Fresh villus tissues were obtained from each of 3 cases in the RSA group and the normal induced abortion group. The tissues were washed with PBS, after which the samples were immediately frozen in liquid nitrogen and used for chemokine detection.

### 2.2 Detection of vaginal flora diversity

The cotton swabs were rinsed in protective solution. Then, 1 mL of protective solution containing the sample was centrifuged at 12,000 rpm for 15 min, and then the supernatant was discarded. Vaginal flora DNA was extracted according to the instructions of the Trace Flora DNA Extraction Kit-I (LS-R-N-007H-50/100, Longsee). The 338F and 806R primers were used, and the sample-specific Barcode sequence was added to amplify the V3–V4 hypervariable region of the 16S rRNA gene (approximately 480 bp). The primer sequences are 338F: 5′-ACTCCTACGGGAGGCAGCA-3′, 806R: 5′-GGACTACHVGGGTWTCTAAT-3′ (barcode sequence not shown). The DNA was amplified by PCR using Q5 High-Fidelity DNA Polymerase (M0491, NEB).

The PCR-amplified product was detected by 2% agarose gel electrophoresis, and the target fragment was subjected to gel recycling using an AxyPrep DNA gel recycling kit (AP-GX-50G, Axygen). According to the preliminary quantitative results of electrophoresis, the recycling product of PCR amplification was subjected to fluorescence quantification using the fluorescent reagent Quant-iT PicoGreen dsDNA Assay Kit (P11496, InvitrogenTM). Quantitative analysis was performed in a microplate reader (BioTek, FLx800). According to the quantitative fluorescence results, each sample was mixed in a corresponding ratio according to each sample's sequencing requirement. The sequencing library was prepared using the TruSeq Nano DNA LT Library Prep Kit (FC-121-4001, Illumina). Prior to sequencing, quality testing of the library was performed on an Agilent Bioanalyzer using an Agilent High Sensitivity DNA Kit (5067-4626, Agilent). A qualified library had a single peak without overlapping regions. The library was then quantified using a Promega QuantiFluor fluorescence quantification system with a Quant-iT PicoGreen dsDNA Assay Kit (P11496, Invitrogen); the qualified library concentration should be over 2 nM. Each qualified sequencing library (index sequence non-repeatable) was diluted by a gradient, mixed according to the required sequencing amount, and then denatured into single strands by NaOH for computer sequencing. The sequencing reagent was MiSeq Reagent Kit V3 (600 cycles) (MS-102-3003, Illumina), and a MiSeq sequencer was used for 2 × 300 bp paired-end sequencing.

### 2.3 Bioinformatic analysis

The raw data for high-throughput sequencing was screened based on sequence quality. Quality testing of the double-ended sequences with the FASTQ format was conducted, one by one, using the sliding window method: the window size was 10 bp, the step size was 1 bp, and the movement started from the first base position of the 5′ end. The average quality of the base in the window was required to be ≥Q20 (that was, the average sequencing accuracy of the base was ≥99%). The sequence was truncated from the first window, whose average quality value was lower than Q20, and the length of the sequence after truncation was required to be ≥150 bp without the existence of a fuzzy base (ambiguous base) N. Afterwards, using FLASH software (v1.2.7, Magoc and Salzberg, 2011), the double-ended sequences that were primed by quality were paired according to overlapping bases: it was required that the length of the overlapping base of the 2 sequences of Read 1 and Read 2 was ≥10 bp, and base mismatching was not allowed. Finally, according to the index information corresponding to each sample (i.e., the Barcode sequence, a small base sequence used to identify the sample at the beginning of the sequence), the connected sequence identification was assigned to the corresponding sample (the Index sequence is required to be completely matched) to obtain an effective sequence for each sample.

The obtained sequences were merged and classified through operational taxonomic units (OTUs), and each OTU's representative sequence was used for status identification and phylogenetic analysis. Merged and individual OTUs classified the previously obtained sequences with 97% sequence similarity, and the highest abundance sequence in each OTU was selected as the representative sequence, using the QIIME software and the UCLUST sequence alignment tool (Edgar, 2010). Then, based on the number of sequences of each OTU contained in each sample, a matrix file (i.e., OTU table) of OTU abundance in each sample was constructed. According to the abundance distribution of OTU in different samples, the diversity level of each sample was evaluated, and the sparse curve was used to reflect whether the sequencing depth was up to standard. For each OTU's representative sequence, the default indexes were used in the QIIME software, and each OTU's taxonomic information was obtained by comparing the OTU representative sequence with the template sequence of the database. The Greengenes database was applied by default to bacteria (Release 13.8, DeSantis et al, 2006).

### 2.4 Chemokine testing

In all, 100 mg of dry ice was ground and processed according to the instructions of the Human Chemokine Array C1 (AAH-CHE-1-2, RayBiotech) kit. The scanning was performed by an ImageQuant LAS4000 Scanner (GE Healthcare Corporate) imaging system with the high-resolution scanning parameter. Data were extracted using the scanner's built-in analysis software.

### 2.5 Data analysis of clinical samples and chemokines

GraphPad Prism 6 software was used to detect chemokines. The measurement data were expressed as the mean ± standard deviation (x ± s) and were analyzed by group *T* test. The enumeration data were expressed by (%), and *X*^2^ was used for testing. *P* < .05 was considered statistically significant.

## 3 Results

### 3.1 Clinical information of the subjects

The clinical information of the URSA patients and the women with normal induced abortions included in this study was statistically analyzed (Table 1). No significant differences were observed in age, BMI, menstrual cycle, pregnancy cycle, or pregnancy between the 2 groups. There was a significant difference in the number of births and abortions. Moreover, there were no abnormalities in the vaginal secretions after routine testing of the normal induced abortion group, and no antibiotics or tocolytic agents were used; the vaginal secretions were detected as abnormal in the URSA group and antibiotics and tocolytic agents were used in this group. In addition, there was no endometritis, amnionitis, cervicitis, urinary tract infections, or other drug use in either group.

**Table 1:** The clinical information of the normal pregnancy induced abortion and unexplained recurrent spontaneous abortion samples.

	Normal pregnancy induced abortion (n = 27)	Unexplained recurrent spontaneous abortion (n = 31)	*P*
Years	31 (23–37)	32 (27–45)	.5
BMI (kg/m^2^)	20.73 (19–22)	20.53 (18.5–22)	.5
Menstrual cycle (d)	29 (28–31)	29 (28–35)	.5
Pregnancy cycle (wk)	7 (5–7)	6 (4–14)	.7
Gravidity	3 (1–6)	4 (2–8)	.1
Maternal	1 (0–3)	0 (0–1)	<.01
Abortion	2 (1–4)	3 (1–7)	<.01
Contraceptive methods	Condom (70.37%)	Condom (0)	1
	Rhythm method (29.63%)	Rhythm method (0)	
Antibiotics	Nifuratel (0)	Nifuratel (83.87%)	<.01
	Canesten (0)	Canesten (9.68%)	
	Azithromycin (0)	Azithromycin (6.45%)	
Tocolytic agent	Progesterone (0)	Progesterone (100%)	<.01
	LMWH (0)	LMWH (100%)	
	Immune globulin (0)	Immune globulin (100%)	
Vaginal secretion	Dysbacteriosis (0)	Dysbacteriosis (35.48%)	<.01
	Bacterial vaginosis (0)	Bacterial vaginosis (48.39%)	
	Colpomycosis (0)	Colpomycosis (9.68%)	
	Mycoplasma+ (0)	Mycoplasma+ (6.45%)	

### 3.2 Test results of vaginal flora diversity

**Figure 2:**
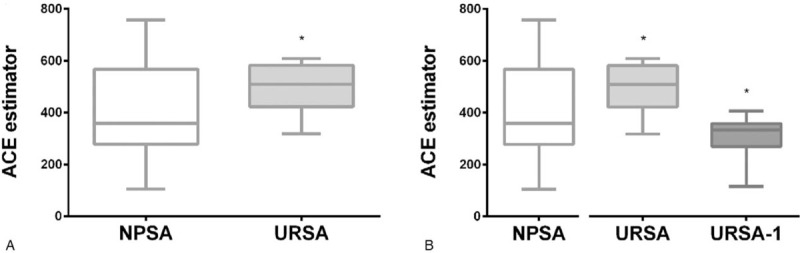
Comparison of ACE diversity index of vaginal flora through Alpha diversity analysis. (A) Regarding the comparison on the ACE abundance estimation index of vaginal flora between the natural pregnancy induced abortion (NPSA) and recurrent spontaneous abortion (URSA), the vaginal flora diversity of recurrent spontaneous abortion is higher than that of natural pregnancy abortion, ^∗^*P* < .05; (B) Regarding the comparison on the ACE abundance estimation index of vaginal flora among natural pregnancy induced abortion (NPSA), recurrent spontaneous abortion (URSA) and patients who have successful fetus protection, the vaginal flora diversity of recurrent spontaneous abortion patients who have successful fetus protection has decreased, ^∗^*P* < .05.

**Figure 3:**
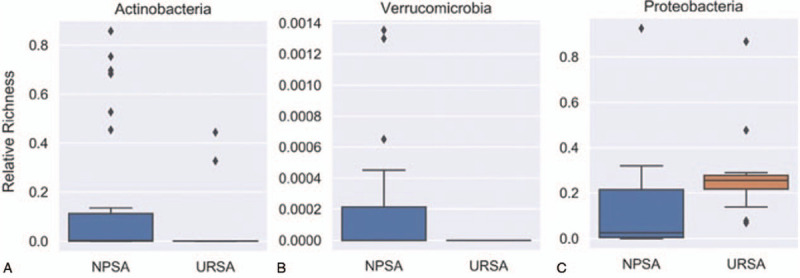
The relative flora abundance of NPSA and URSA groups on phylum level. A. Actinobacteria of NPSA group has higher expressive abundance compared with URSA group. B. Verrucomicrobia of NPSA group has higher expressive abundance compared with URSA group. Proteobacteria of URSA group has higher expressive abundance compared with NPSA group.

Firstly, the 16s rRNA sequencing assay was used to detect vaginal flora in 27 women with spontaneous abortions and 31 women with URSAs in southern China to infer the vaginal flora gene and species function. As shown in Figure 2, the alpha population diversity in URSA patients is higher than that of NPSA patients. Further, the differences in vaginal flora in the NPSA and URSA groups were detected by LEfSe analysis. As shown in Figure 3, the logarithmic scores of the LDA difference analysis are used to describe the expressive abundance of flora with significant differences between groups. Highly expressed flora in the NPSA group include Actinomycetes, Bifidobacteria, *Gardnerella*, *Veillonococcus*, *Megacoccus*, Peptostreptococcaceae, Comamonadaceae, Anaerolineae, Rhodospirillaceae, and Verrucomicrobiaceae, with an LDA score >2; highly expressed flora in the URSA group include Gammaproteobacteria, Proteobacteria, *Pseudomonas*, *Moraxella*, *Ruminococcus*, *Collinsella aerofaciens*, Alteromonadaceae, *Cellvibrio*, *Arthrobacter*, *Roseburia*, and Micrococcaceae, with an LDA score >2. The classification level tree, displayed by the cladogram, describes the hierarchical relationship of all flora from phylum to genus (from the inner circle to the outer circle) in the sample community, and the average relative abundance of the corresponding flora. On the phylum level, the differential flora mainly includes Actinobacteria, Proteobacteria, and Verrucomicrobia. On the genus level, the differential flora mainly includes *Pseudomonas*, *Gardnerella*, *Bifidobacterium*, *Megacoccus*, *Akkermansia*, *Roseburia*, *Collinsella aerofaciens*, *Arthrobacter*, *Ruminococcus*, and *Cellvibrio*, among which *Pseudomonas*, *Roseburia*, *Collinsella aerofaciens*, *Arthrobacter*, *Ruminococcus*, and *Cellvibrio* have high expressive abundance in the URSA group, while *Gardnerella*, *Bifidobacterium*, *Megacoccus*, and *Akkermansia* have high expressive abundance in the NPSA group.

**Figure 4:**
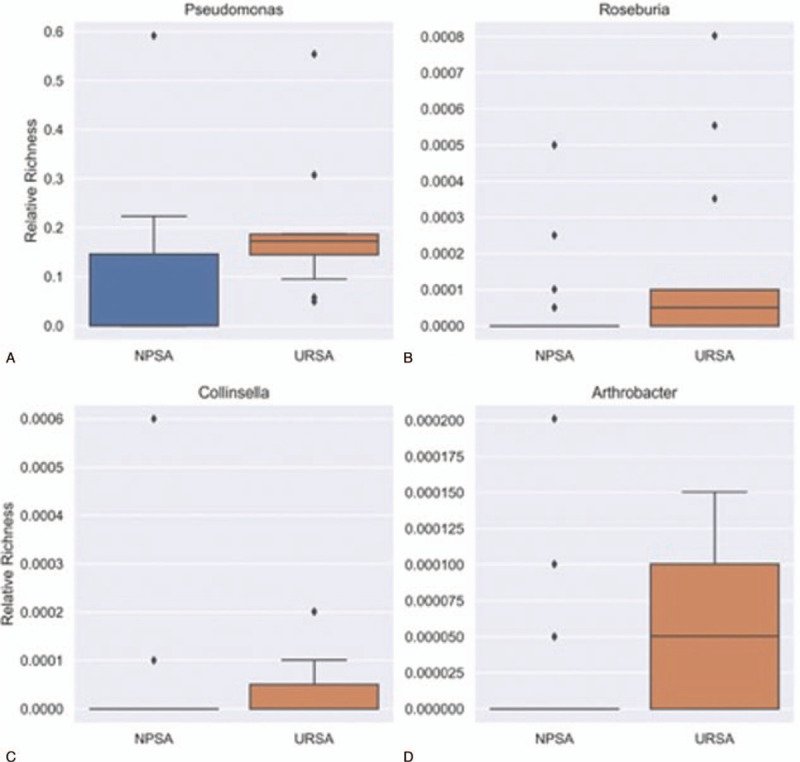
The relative flora abundance of NPSA and URSA groups on genus level. A, B, C, D. Expressive abundance of Pseudomonas, Roseburia, Collinsella aerofaciens, and Arthrobacter is higher in URSA group than NPSA group.

**Figure 5:**
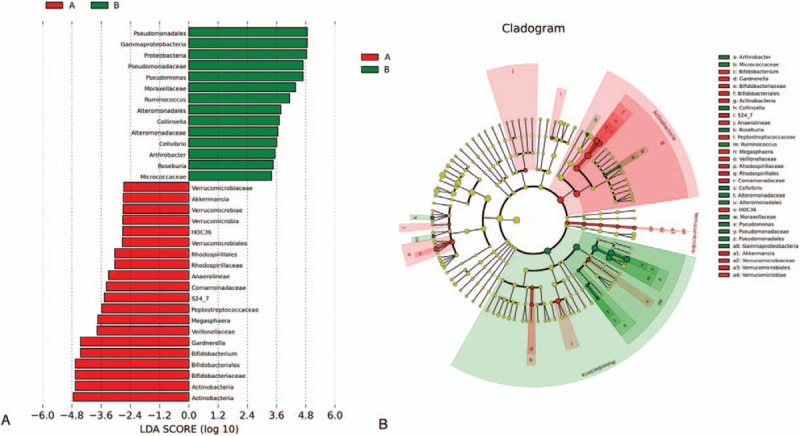
LEfSe analysis of vaginal flora differences in NPSA and URSA groups. A. logarithmic scores of LDA difference analysis are used to describe the expressive abundance of flora with significant differences between groups. High expressive flora in NPSA group include: Actinomycetes, Bifidobacteria, Gardnerella, Veillonococcus, Megacoccus, Peptostreptococcaceae, Comamonadaceae, Anaerolineae, Rhodospirillaceae, Verrucomicrobiaceae, LDA score>2; High expressive flora in URSA group include: Gammaproteobacteria, Proteobacteria , Pseudomonas, Moraxella, Rumenococcus, Collinsella aerofaciens, Alteromonadaceae, Cellvibrio, Arthrobacter, Roseburia, Micrococcaceae, LDA score>2. B. The classification level tree displayed by cladogram describes the hierarchical relationship of all the flora from phylum to genus (from the inner circle to the outer circle) in the sample community, and the average relative abundance of the corresponding flora. On phylum, the differential flora mainly includes Actinobacteria, Proteobacteria, and Verrucomicrobia. On genus, the differential flora mainly includes Pseudomonas, Gardnerella, Bifidobacterium, Megacoccus, Akkermansia, Roseburia, Collinsella aerofaciens, Arthrobacter, Ruminococcus, and Cellvibrio, among which the Pseudomonas, Roseburia, Collinsella aerofaciens, Arthrobacter, Ruminococcus, and Cellvibrio have high expressive abundance in URSA group and the Gardnerella, Bifidobacterium, Megacoccus, and Akkermansia have high expressive abundance in NPSA group.

The differences in vaginal flora in the NPSA and URSA groups on the phylum and genus levels were also determined (Figs. 4 and 5). Actinobacteria has a higher expressive abundance in the NPSA group compared with the URSA group. Verrucomicrobia has a higher expressive abundance in the NPSA group than the URSA group. Proteobacteria has higher expressive abundance in the URSA group than the NPSA group. The expressive abundance of *Pseudomonas*, *Roseburia*, *Collinsella aerofaciens*, and *Arthrobacter* is higher in the URSA group than the NPSA group.

**Figure 6:**
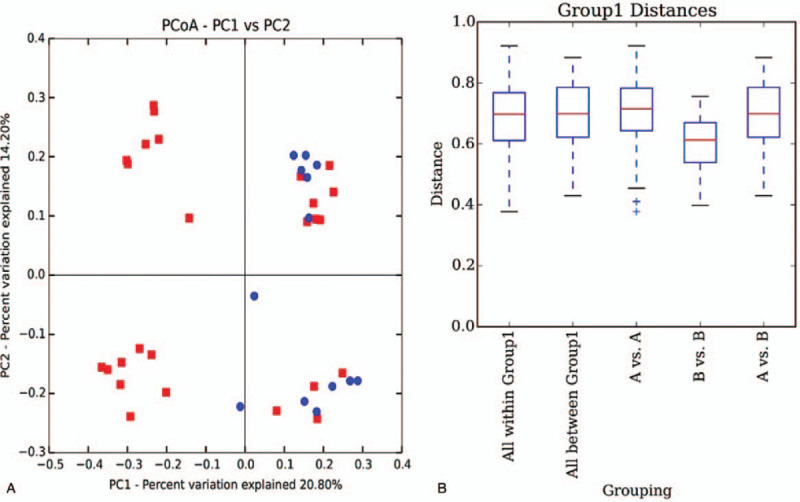
(A) By using Unweighted UniFrac PCoA analysis, each dot represents a sample with the red ones representing NPSA patients and blue ones representing URSA patients; most NPSA samples can be separated from URSA samples, indicating a certain difference of sample composition of the 2 groups. (B) Box plot of Unweighted UniFrac distance of multiple groups. (A) stands for NPSA group and (B) for URSA group. The difference in (A) vs (B) is significantly higher than the difference within (B) group, indicating the statistical difference between NPSA and URSA samples.

Figure 6 shows the distribution of vaginal flora in both patient groups using unweighted UniFrac PCoA and unweighted UniFrac analysis. Most NPSA samples can be separated from URSA samples, which indicate a certain difference in sample composition of the 2 groups. The difference in both groups is significantly higher than the difference within the B group, which indicates a statistical difference between NPSA and URSA samples.

### 3.3 Test results of chemokines

An ImageQuant LAS4000 Scanner imaging system was used to scan the chemokine chip. The size and brightness of the dot reflected the chemokine expression level. The larger and brighter the dot, the higher the expression of the chemokine, while the smaller and dimmer the dot, the lower the expression. The expression levels of CCL4/CCL8/CCL3/CCL5/CCL2 in the NPSA group are 1725.38 ± 1.24, 493.97 ± 0.82, 729.54 ± 0.65, 16157.81 ± 2.58, and 3589.14 ± 2.23, respectively, and the expression levels of CCL4/CCL8/CCL3/CCL5/CCL2 in the URSA group are 10261.18 ± 2.24, 2415.07 ± 1.79, 2025.72 ± 1.34, 30690.75 ± 2.86, and 5129.93 ± 1.62, respectively, which indicates that the expression levels of CCL4/CCL8/CCL3/CCL5/CCL2 are higher in URSA than in NPSA (Fig. 2, *P* < .05). The expression levels of CCL4/CCL8/CCL3/CCL5/CCL2 are 5.95 ± 0.56, 4.89 ± 0.62, 2.78 ± 0.58, 1.90 ± 0.46, and 1.43 ± 0.80, respectively, times higher in URSA compared with the NPSA group.

## 4 Discussion

Thus far, preventing and treating RSA have presented difficult challenges from a clinical perspective.^[1,2]^ Its potential negative influence on reproductive health has brought instability to the families of the patients as well as to society. Although our understanding of this disease's pathogenesis is thorough, due to its numerous causes, mixed factors, and complex relationships, the clinical diagnosis and treatment of this disease has not improved significantly. More seriously, the incidence rate of RSA has increased significantly in recent years (Fig. 1), and the number of URSA cases has also increased.^[3,16]^ Therefore, it is particularly important for us to control the incidence of RSA by clarifying the pathogenesis of RSA, finding potential therapeutic targets and new diagnostic markers, as well as developing new methods and treatments for early diagnosis and intervention.

**Figure 7:**
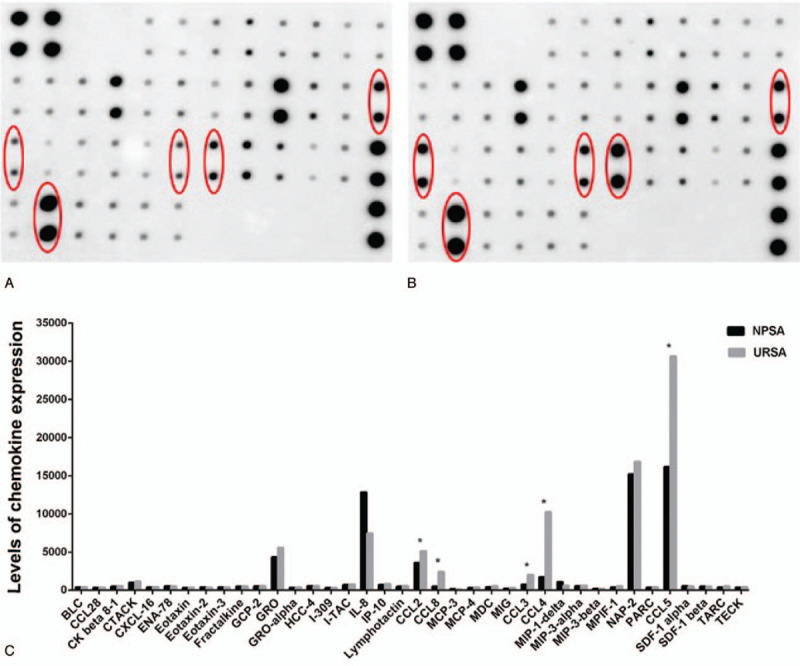
The scanning result of chemokine chip. (A) The chemiluminescence detection results of chemokine in NPSA group; (B) The chemiluminescence detection results of chemokine in URSA group. The size and brightness of the dot reflected the expression level of chemokine. The larger and the brighter of the dot, the higher the expression of chemokine, and the lower on contrast. From left to right, the red circle marks the expression quantity of CCL8/CCL5/CCL3/CCL4/CCL2 and the expression quantity of chemokine in URSA group in higher than NPSA. (C) The comparison of the chemokine expression quantity between NPSA group and URSA group. The expression quantity of CCL2/CCL8/CCL3/CCL4/CCL5 in URSA is significant higher than that of NPSA (∗*P* < .05).

In this study, 16s rRNA sequencing was used to detect vaginal flora in 27 women with spontaneous abortion and 31 women with URSA in southern China to determine the vaginal flora gene and species function. Then, using a chemokine protein chip, the mechanism of the effects of vaginal flora dysregulation on the local immune system of patients with RSA was clarified (Fig. 7). From the ACE diversity index box plot, we found that the vaginal flora of patients with RSA has a higher alpha diversity than that of patients with natural abortion during pregnancy (Table 1). Moreover, we found that women with RSAs often have vaginal infections, such as BV, fungal vaginosis, or mycoplasma infection (Table 1). As stated in a report on BV,^[32]^ vaginal flora diversity will increase after such infectious diseases occur. PCoA analysis results showed that, based on unweighted UniFrac distance, the vaginal flora of patients with RSA is significantly different from that of patients with spontaneous pregnancy abortion, which can explain the significant structural differences between the 2 groups. It is also interesting to note that, on the phylum level, the Proteobacteria in URSA group has higher expressive abundance than in the NPSA group, and on the genus level, *Pseudomonas* is also a bacterial genus with a high expressive abundance. In addition, *Pseudomonas* belongs to the Proteobacteria phylum. *Pseudomonas* is not part of the vaginal flora; rather, it is a gram-negative bacillus that is also an opportunistic pathogen.^[33]^ This pathogen can often cause acquired infections in hospitals and produce a variety of toxic substances. *Pseudomonas* infection, which can occur in any part of the body and in any tissue, is common in burns or wounds, in the middle ear, cornea, urethra, and respiratory tract. This bacterium can also cause endocarditis, gastroenteritis, empyema, and even sepsis.^[34]^

The clarification of the difference between the vaginal flora of URSA patients and that of women with NPSA has provided important significance for the early prevention and diagnosis of this disease in the clinic. In the screening and analysis of these differential flora, gram-negative bacteria (such as *Pseudomonas*) in the Proteobacteria phylum and endotoxin are the main components of the cell wall of this flora,^[35]^ and their abnormal expression affects macrophages, which can then activate the immune system and release chemokines.^[36]^ The protein chip results of the chemokine analysis has also validated this assumption with the observation of the significant increase in the expression of CCL2, CCL3, CCL4, CCL5, and CCL8 in villus tissues of women with URSA (Fig. 7B). As markers of white blood cells, these chemokines can recruit a large number of monocytes, neutrophils, and other effector cells and promote their concentration in the vagina,^[27,37,38]^ which inhibits reproduction of these harmful vaginal bacteria.

The results of this study initially suggest a significant correlation between vaginal microbial flora imbalance and URSA, with vaginal *Pseudomonas* as a potential diagnostic marker for a new type of RSA. The results have also shown that the pathogenesis of URSA may be caused by an imbalance of the patients’ vaginal flora (especially that of *Pseudomonas*); infections caused by flora imbalances can be transmitted to the uterus through the vagina, where they can activate chemokines to induce a local immune response. This, in turn, will cause microcirculation disturbances of the local immune system and lead to RSA. Abnormal expression of the vaginal flora may become a new target that can provide important theoretical guidance for further improvement in early URSA diagnosis and treatment. Admittedly, this study also has some limitations, which need to be improved further. For example, the strains of vaginal *Pseudomonas* that play the key role in regulation should be determined. How do these flora cause vaginal and intrauterine infection, and how do they affect and regulate chemokine expression? How does *Pseudomonas* translocate into the uterus and induce infection or immune regulation? These issues still require further study. In summary, the results of our study have provided a new research perspective for diagnosis and prevention of URSA; hopefully, this can become a new theoretical basis for the prevention and treatment of this disease.

## Author contributions

**Conceptualization:** Zhu-Lin Miao.

**Data curation:** Tao Fan, Xing-Ming Zhong, Xiang-Cai Wei, Heng Cheng.

**Formal analysis:** Xing-Ming Zhong, Zhu-Lin Miao.

**Funding acquisition:** Qing Xiao.

**Investigation:** Zhu-Lin Miao, Si-Ying Luo, Heng Cheng.

**Methodology:** Xiang-Cai Wei, Si-Ying Luo.

**Resources:** Xiang-Cai Wei.

**Writing – original draft:** Tao Fan, Qing Xiao.

**Writing – review & editing:** Qing Xiao.
